# Lung-Protective Effect of Alveolar Recruitment Maneuvers in Children with Right Vertical Infra-Axillary Thoracotomy for Repair of Congenital Heart Disease

**DOI:** 10.3390/children13050588

**Published:** 2026-04-24

**Authors:** Liang Zhang, Chengbin Wang, Chen He, Xiaonan Wang, Lin Lin, Jun Ma, Sheng Wang

**Affiliations:** Center for Anesthesiology, Beijing Anzhen Hospital, Capital Medical University, Beijing Institute of Heart Lung and Blood Vascular Diseases, Beijing 100000, China

**Keywords:** alveolar recruitment maneuver, atrioventricular septal defect, oxygenation, pulmonary complication

## Abstract

**Highlights:**

Our findings indicate that the use of ARM improves the oxygenation profile and attenu-ates the systemic inflammatory response after extracorporeal circulation. In addition, ARM reduces postoperative pulmonary complications in children with a common congenital heart defect corrected via RVIAT.

**What are the main findings of this study?**

• ARM improves the oxygenation profile.
• ARM reduces the incidence of pulmonary atelectasis in children.

**What are the implications of the main findings?**

• ARM reduces the postoperative mechanical ventilation time.
• Children’s hemodynamic status remains stable during ARM.

**Abstract:**

Background/Objectives: Right vertical infra-axillary thoracotomy to repair ventricular septal defects (VSDs) and atrial septal defects (ASDs) is less invasive than conventional surgical repair via median sternotomy. However, right vertical infra-axillary thoracotomy (RVIAT) may result in unilateral lung injury, a serious postoperative complication requiring extracorporeal circulation and unilateral lung collapse. The aim of this study was to evaluate whether repeated lung recruitment provides enhanced respiratory compliance and lung oxygenation in children who have undergone right vertical infra-axillary thoracotomy (RVIAT) to correct a congenital heart defect. Methods: Eligible participants were children with a common congenital heart defect corrected via RVIAT. Seventy-seven children were randomly classified into two groups. In group A (*n* = 39), an alveolar recruitment maneuver (ARM) was performed immediately after cardiopulmonary bypass. Children in group C (*n* = 38) did not receive any additional interventions during surgery. Results: The ARM group tolerated open lung ventilation without significant hemodynamic instability. Compared to controls, intraoperative PaO_2_, PaO_2_/FiO_2_ and lung compliance (Com_dyn_) improved in group A (*p* < 0.05), who also showed a significantly lower IL-6 (*p* < 0.05). In addition, group A had a lower incidence of lung injury and lung atelectasis than Group C at specific post CPB time points. Conclusions: Our findings provide some indication that the application of ARM could effectively improve the oxygenation profile, reduce postoperative pulmonary complications, and attenuate the postoperative inflammatory response in children with a common congenital heart defect corrected via the RVIAT technique.

## 1. Introduction

Impaired lung function after cardiopulmonary bypass (CPB) is one of the most common complications following cardiac surgery and is associated with a higher risk of morbidity and mortality [1]. Due to definition and population differences among various studies, the incidence of hypoxemia in adults can range as high as 20–25% [2]. After cardiac surgery with CPB, children may experience significant pulmonary complications due to increased systemic inflammatory response and resulting atelectasis. These complications are more commonly observed after CPB than after the use of anesthesia alone [1].

Minimal right vertical infra-axillary thoracotomy (RVIAT) is performed to correct atrial septal defects (ASDs) and ventricular septal defects (VSDs), as it is safe and less invasive than the standard approach, median sternotomy. However, because the procedure requires cardiopulmonary bypass (CPB) and unilateral lung collapse, postoperative unilateral lung injury may occur, with a reported frequency between 2.1 and 25% [3]. Furthermore, increased pulmonary blood flow and/or pressure are associated with pulmonary edema formation and reduced pulmonary compliance [4]. Accordingly, pulmonary dysfunction is a frequent postoperative complication of minimally invasive cardiac surgery (MICS).

‘Open lung’ ventilation is commonly used in patients with acute lung injury and has been shown to improve intraoperative oxygenation in patients undergoing general anesthesia [5]. Intra-operative lung-protective ventilation is recommended by international consensus as a key intervention for preventing postoperative pulmonary complications [6]. However, the effect of alveolar recruitment maneuver (ARM) and lower tidal volumes with positive end-expiratory pressure (PEEP) in children under general anesthesia during RVIAT remains unclear.

This study was performed to determine whether repeated lung recruitment improves respiratory compliance and lung oxygenation in children undergoing congenital heart disease (CHD) correction with right vertical infra-axillary thoracotomy.

## 2. Methods

We performed a randomized, prospective, controlled clinical study, which conformed to the principles outlined in the Declaration of Helsinki and received approval from the Medical Ethics Committee at Beijing Anzhen Hospital, Capital Medical University. Written informed consent was obtained from the parents of all children prior to their inclusion. This study is registered in the www.chictr.org.cn database (ID: ChiCTR1900023576).

### 2.1. Subjects

One hundred children with American Society of Anesthesiologists (ASA) scores between class II and III who underwent congenital heart disease correction with right vertical infra-axillary thoracotomy under general anesthesia participated in this randomized controlled study. The inclusion criteria were an age > 6 months and <3 years, a preoperative diagnosis of atrial septal defect (ASD), ventricular septal defect (VSD), or partial atrioventricular septal defects (PAVSD). The exclusion criteria were a refusal to give consent, BMI < 18 kg/m^2^ or >35 kg/m^2^, significant cardiac dysfunction (left ventricular ejection fraction of 40%), asthma requiring bronchodilator therapy, pulmonary infection, and emergency surgery.

An independent research assistant performed randomization with an online tool (https://www.sealedenvelope.com/randomisation/ (accessed on 10 January 2020)) to allocate 100 children (23 were excluded) into a control group (group C, *n* = 38) and an alveolar recruitment maneuver group (ARM, group A, *n* = 39). Clinicians and radiologists assessing postoperative pulmonary complications (especially chest radiographs for atelectasis and other lung findings) were blinded to group assignment. In group A, immediately after cardiopulmonary bypass, lung recruitment (automated with the anesthesia machine) was achieved by sequential increases in PEEP in three steps from 10 cm H_2_O (for 3 breaths) to 15 cm H_2_O (for 3 breaths) and 20 cm H_2_O PEEP (for 3 breaths). Following ARM, volume control was established using Vt 6 mL/kg and reductions in PEEP to 10 cmH_2_O. The ARM lasted about 30–60 s and was repeated at 30 min after the first recruitment. Group C did not receive any additional interventions during surgery.

### 2.2. Anesthesia

Standard monitoring was performed for all children. Anesthesia was induced with sufentanil (1 µg/kg), midazolam (0.2 mg/kg), and pipecuronium bromide (0.1 mg/kg) and maintained with a hypnotic (propofol), pipecuronium bromide, and an opioid (sufentanil). Patient monitoring included continuous 5-lead electrocardiographic registration with ST-segment analysis, peripheral oxygen saturation by pulse oximetry, radial arterial blood pressure, central venous pressure, capnography, rectal temperature, and urine output. The radial artery catheter was connected to a monitor for pulse contour analysis (MostCare Up Monitor, Vygon, Ecouen, France), and the resulting signal was processed for determination of hemodynamic variables.

### 2.3. Surgical Technique

The children received volume-controlled mechanical ventilation using a tidal volume (VT) of 6–8 mL/kg of body weight, an inspiratory/expiratory time ratio (I: E) of 1:1.5, and an O_2_/air mixture (FiO_2_ of 0.5) administered at 2 L/min. The respiration rate was adjusted to maintain an end-tidal CO_2_ tension of 35 to 40 mmHg. At the end of the surgery, a recruitment maneuver (manually applying a continuous positive airway pressure of 30 cm of water for 30 s) was repeated 3 times for lung re-expansion after one-lung ventilation (OLV) to avoid atelectasis in group C. Children in group A received more lung recruitment procedures, including maintaining a higher PEEP.

The children were canted to the right at an angle of 60°. The right arm was placed on the head with a 120° abduction from the shoulder. Skin incision was started from the third intercostal space in the midaxillary line and continued until the fifth intercostal space. The right lung was pushed back with a wet sponge and a malleable retractor. As a result, the intraoperative operation caused the right lung to be trapped.

The surgeon placed standard purse sutures on right side of the ascending aorta, the superior vena cava, and the right aortocaval junction. After heparinization, the surgeon cannulated the ascending aorta, the superior vena cava and the inferior vena cava. Then, a standard CPB was initiated, and aortic cross-clamping was performed in the same incision. The same VSD or ASD closure performed in all procedures. Finally, the pericardium was partially closed, and a chest drain was placed in the right thorax.

### 2.4. CPB Management

The dose of heparin used for anticoagulation during CPB was 300 U/kg plus additional doses to achieve and maintain an activated clotting time of more than 480 s.

During CPB, a minimum rectal temperature of 30–32 °C was maintained. Management of CPB included alpha-stat pH management, MAP in the range of 50–80 mmHg, hematocrit of 20–25%, and a non-pulsatile flow rate of 2.0–2.4 L/min/m^2^. Protamine sulfate was used to reverse heparin-induced anticoagulation after separation from CPB.

### 2.5. Postoperative Care

All children were transferred to the ICU after cardiac surgery, where they underwent management according to institutional protocols. Volume–pressure-controlled ventilation was applied with a tidal volume of 6–8 mL/kg, PEEP of 5 to 7 mmHg, FiO_2_ of 50% and respiratory rate of 10 to 25/min to maintain an end-tidal CO_2_ tension of 35 to 45 mmHg. Pressure support ventilation with a 10 cmH_2_O pressure support level and 5 cmH_2_O PEEP was performed upon recovery of spontaneous respiration.

The extubation criteria were a respiratory rate of 15 to 30 breaths/min, heart rate within 20% of baseline for at least for 1 h, PaO_2_ of >70 mmHg, PaCO_2_ of <48 mmHg, and pH of 7.35 to 7.50 upon arterial blood gas analysis.

### 2.6. Primary and Secondary Outcomes

The primary outcomes measured were arterial blood gas analysis, peak-inspiratory pressures (PIP), lung compliance (Com_dyn_), and PaO_2_/FiO_2_. Arterial blood gas analysis was calculated at the following time points: 5 min prior to incision (T1), 3 min after the first ARM (T2), 3 min after the second ARM (T3), and 2 h after weaning from CPB (T4). Global hemodynamic variables (HR, MBP, and CI) were recorded at regular intervals: T1, T2, T3, and T4. In all groups, the same measurements were recorded at the same time points. These indicators were chosen a priori because they are essential for postoperative recovery of lung function.

The secondary outcomes were hemodynamic indicators (HR, MBP, and CI) mechanical ventilation time, pulmonary collapse, hospital mortality, duration of ICU and postoperative hospital stays, and postoperative lung complications (atelectasis, pulmonary edema, pneumonia, pleural effusion, and acute lung injury), which were recorded after surgery. Pulmonary complications were diagnosed according to clinical symptoms and/or radiographic evidence (X-ray images) according to the criteria for congenital heart disease [7,8], including chylothorax, pleural effusion, pneumonia, pneumothorax, atelectasis, postoperative respiratory insufficiency requiring mechanical ventilatory support > 7 days, postoperative respiratory insufficiency requiring reintubation, and postoperative respiratory failure requiring tracheostomy.

In addition, the children’s plasma levels of IL-6 and TNF-α were determined at T1 and T4. Blood samples were drawn into tubes containing ethylenediaminetetraacetic acid via the central venous catheter and centrifuged at 3000 rpm for 20 min. The extracted plasma was stored in polypropylene tubes at −80 °C until analysis for IL-6 and TNF-α using a quantitative sandwich enzyme immunoassay (Quantikine ELISA; R&D Systems, Inc., Minneapolis, MN, USA). All enzyme immunoassays were performed with a V-MAX 220 VAC ELISA reader (Molecular Devices, Palo Alto, CA, USA).

### 2.7. Statistical Analysis

Descriptive analysis was performed using the mean ± SD for continuous variables and frequencies (percentages) for categorical data. For comparisons of categorical variables, Chi-square analysis was performed. Student’s t-test was used to compare the means of two samples. The arterial blood gas variables were analyzed using repeated-measures analysis of variance (ANOVA). All data collected were stored electronically and analyzed using SPSS 17.0 software (SPSS Inc., Chicago, IL, USA). Kolmogorov–Smirnov and normal quantile plots were conducted to determine whether the continuous variables were normally distributed. Graphics were produced using GraphPad Prism 5 (GraphPad Software, San Diego, CA, USA).

## 3. Results

### 3.1. Patient Characteristics

**Figure 1:**
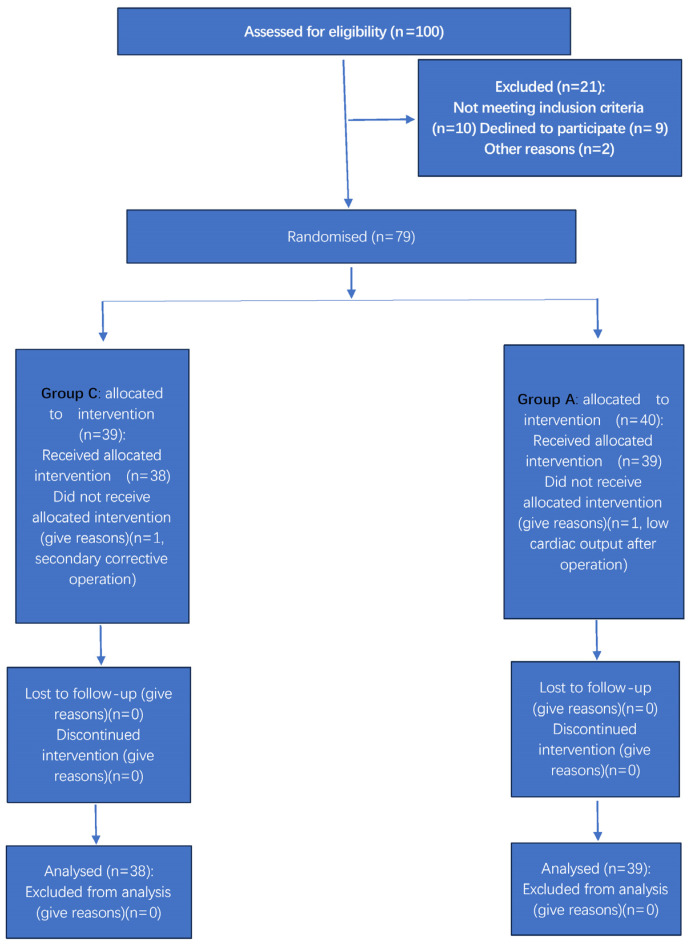
CONSORT diagram of the sub-study. PEEP—positive end-expiratory pressure; ARM—recruitment maneuver.

In total, 100 children were assessed for eligibility, and 23 were excluded before randomization. The final analysis included 77 children after excluding 2 children with a secondary corrective operation and low cardiac output after the operation (group C, *n* = 38; group A, *n* = 39). (Figure 1).

The 77 children (44 male and 33 female; median age, 1.4 years; median weight, 8.3 kg) had a ventricular septal defect (VSD) (54 children), atrial septal defect (ASD) (10 children), or partial atrioventricular canal (PAVC) correction (13 children). Baseline characteristics were similar between the two study groups (Table 1).

### 3.2. Pulmonary Complication Outcome

Twelve children in the control group and five in group A developed pulmonary complications (*p* = 0.020), with atelectasis detected on chest radiogram and requiring intervention being the most common complication. In addition, children in group A had a lower mechanical ventilation time and postoperative hospital stay than children in group C (Table 2).

### 3.3. Breathing Pattern and Gas Exchanges

**Figure 2:**
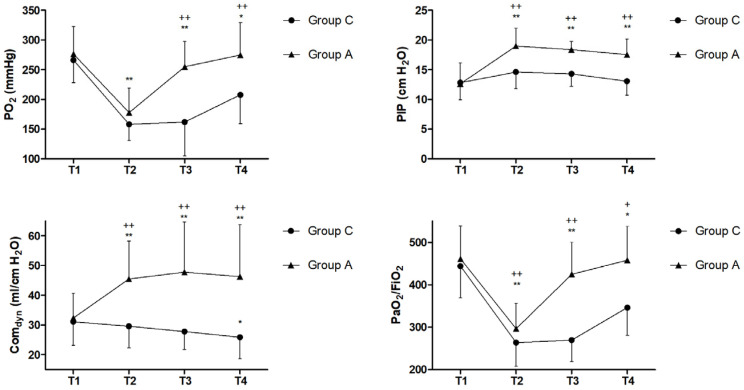
Changes in PO2 (mmHg), OI, Com_dyn_ (mL/cm H_2_0), and PIP (cmH_2_O) at different time points. * *p* < 0.05, ** *p* < 0.01, compared with T1; ^+^ *p* <0.05, ^++^ *p* < 0.01, between the groups. T1, 5 min prior to incision; T2, 3 min after the first ARM; T3, 3 min after the second ARM; T4, 2 h after weaning from CPB.

After cardiopulmonary bypass, PaO2 and PaO_2_/FiO_2_ decreased significantly (*p* < 0.05); however, the ARM improved intraoperative PaO2 (*p* < 0.05) after the intervention. Because of the higher PEEP used, the peak inspiratory pressures (PIPs) and lung compliance (Com_dyn_) were significantly higher in the ARM group compared with group C (Figure 2).

### 3.4. Intraoperative Proinflammatory Cytokine

There were no significant differences in plasma concentrations of IL-6 and TNF-α between the two groups at the very beginning of the operation (*p* > 0.05). However, at 2 h after weaning from CPB, IL-6 was lower in group A than in group C, and the difference was statistically significant (*p* < 0.05) (Table 3).

### 3.5. Hemodynamics

During the ARM (Table 4) in group A, the cardiac index and mean blood pressure decreased by approximately 15% compared with the control group (*p* < 0.005), returned to previous values immediately after the ARM, and remained stable thereafter. The heart rate remained stable throughout the procedure.

## 4. Discussion

We encountered a case of severe post-cardiopulmonary bypass hypoxemia in a child with ventricular septal defect repair. The child’s oxygen saturation dropped to 30–40% and then required a second extracorporeal circulation (as opposed to a pulmonary hypertension crisis or tracheal tube obstruction). Pulmonary insufficiency after CPB is related to impaired oxygenation due to remarkable and transient increases in atelectasis, intrapulmonary shunting, and extravascular pulmonary edema during CPB. Unlike in adult patients, there are no well-known methods that can effectively ameliorate hypoxemia after CPB in children with CHD.

The aim of this study was to investigate the outcomes of mechanically regulating lung recruitment to improve lung function recovery in children undergoing cardiac surgery. In this study, the rate of incidence of pulmonary complications in children after cardiac surgery was 31.6%, and performing an ARM after extracorporeal circulation shutdown not only decreased the incidence of pulmonary atelectasis but also improved arterial oxygenation. Moreover, the ARM reduced IL-6 expression and inhibited the inflammatory response. An ARM can reduce the duration of mechanical ventilation time and postoperative hospital stay in children. In accordance with our findings, an ARM can partially improve lung function recovery after extracorporeal circulation in children undergoing RVIAT.

Atelectasis occurs in about 90% of all anesthetized patients [9]. Up to 15–20% of the lung is regularly collapsed at its base during uneventful anesthesia prior to any surgery [10]. In this study, the right lung was pushed back with a wet sponge and a malleable retractor during RVIAT. Mechanical factors may promote the occurrence of lung atelectasis.

In surgical patients, the clinical presentation of lung atelectasis varies from hypoxemia to prolonged mechanical ventilation time and even acute respiratory distress syndrome (ARDS). An ARM is a ventilator maneuver performed to revert atelectasis by means of a brief, controlled increase in airway pressure [11]. Some studies have shown that, during general anesthesia for non-cardiac surgery, ARMs could reduce atelectasis, increase arterial oxygenation, and decrease pulmonary shunting [12,13]. Tusman et al. also demonstrated that a cycling ARM combined with an adequate level of PEEP improved lung physiology in thoracic surgery with one-lung ventilation [14,15]. However, the relationship of ARMs and lower tidal volumes with PEEP in children under general anesthesia during RVIAT remains unclear. In accordance with our findings in children undergoing congenital heart disease surgery, compared with the control group, children in group A demonstrated a significantly reduced incidence of postoperative atelectasis and duration of postoperative mechanical ventilation time.

Zupancich et al. demonstrated that the application of lung-protective ventilation can prevent recurrent alveolar collapse and reopening. Additionally, overinflation of the lung has been shown to be related to improved pulmonary function and decreased concentrations of proinflammatory cytokines in the bronchoalveolar lavage fluid and plasma of patients undergoing cardiac surgery [16,17]. The findings of this study indicate that lung-protective ventilation alone was effective at reducing the expression of inflammatory factors in plasma.

Patients with increased pulmonary blood flow (PBF) show significant abnormalities in respiratory mechanics before surgery, exhibiting decreased dynamic compliance and increased respiratory resistance [18]. Manuela et al. demonstrated that in children with CHD, lung mechanics, PaO_2_/FiO_2_, and lung epithelial lining fluid (ELF) biomarkers were significantly affected by CPB, according to preoperative pulmonary hemodynamics [19].

Furthermore, post-cardiopulmonary bypass hypoxemia typically develops in the first 2 h after CPB in pediatric congenital heart disease surgery [20]. Moreover, as these children require prolonged mechanical ventilation in the intensive care unit after CPB surgery for CHD, their situation differs considerably from that during surgery. In this study, we only targeted the intraoperative period for intervention. Our study demonstrates that in group A, demonstrated better alveolar aeration (PaO_2_, PaO_2_/FiO_2_) and dynamic compliance (Com_dyn_) from after the intervention to 2 h after the operation (T2–T4) than the children in C group. Our findings are similar to the results of Sun et al.’s analysis on the effects of lung-protective ventilation in infants undergoing congenital heart disease surgery [21].

The pathogenesis of CPB-mediated lung injury has been studied extensively. It has been proposed that the inflammatory response to CPB [22,23] and ischemia–reperfusion [24,25] are frequent causes. Such conditions may result in the leakage of fluid into the lungs, a reduction in surfactant activity, a decrease in compliance, a reduction in the functional residual capacity, and an increase in ventilation–perfusion mismatch [26]. In particular, in pediatric cardiac surgery, neonates and infants are exposed to CPB circuits with a significantly large circuit size-to-patient size ratio. Due to their elevated metabolic demand, children are exposed to elevated levels of pump flow in comparison with other age demographics, resulting in greater blood exposure to the CPB circuit’s foreign surface [26]. It can be demonstrated that higher pump flow rates result in elevated shear stress to the vasculature. Furthermore, it is likely that these infants and neonates are subjected to blood-primed CPB, which may contribute to lung injury. The combined effect of these factors may increase the risk of lung injury in neonates and infants. In addition, respiratory burst and inflammatory activity are triggered when oxygen is supplied during re-expansion (and concomitant reperfusion) of the lung.

The contact of blood components with the artificial surface of the CPB machine stimulates and activates neutrophils. This activation is exacerbated by pro-inflammatory mediators such as IL-1, -6, -8, and TNF-a. The main experimental indicator of the present study was a comparison of TNF-α and IL-6 levels before and after ARMs in children, in order to elucidate the inflammatory mechanisms underlying non-infected lung injury. NF-κB, an oxidative stress-sensitive transcription factor, is central to the regulation of the inflammatory response. After activation, NF-κB can regulate the expression of a series of inflammatory cytokines, participate in the development of pulmonary inflammation, and induce the production of TNF-α, IL-1, IL-6, IL-8 and other inflammatory factors, forming a “cytokine cascade reaction” and initiating lung inflammation [27]. Our study demonstrated that in children undergoing congenital heart disease surgery, ARM pretreatment resulted in reduced expression of pro-inflammatory cytokine (IL-6) at 2 h after weaning from CPB. However, we did not observe any significant changes in the level of TNF-α, necessitating further research.

Despite the increase in airway pressure during ARMs, the children’s hemodynamic status remained essentially stable. In group A, the cardiac index was only transiently decreased (approximately 15% compared with the other groups) by the ARM itself (receiving a high degree of PEEP) and returned to previous values immediately after the ARM. A explanation for the optimization of hemodynamic status is that, before the ARM, all blood remaining after extracorporeal circulation was returned to the children through the aortic cannula. Our results agree with those of Cinnella and colleagues [26], who observed changes in hemodynamic status during the recruitment maneuver and with positive end-expiratory pressure in patients undergoing laparoscopic surgery.

The limitations of the current study are as follows: First, our study demonstrated that mechanical ventilation time and number of postoperative pulmonary complications in the ARM group were favorable, but the number of children studied is not sufficient to draw conclusions. Second, we applied a standardized open-lung strategy consisting of an ARM followed by a PEEP of 10 cmH_2_O. Although the data show that this strategy reduced atelectasis and improved in lung oxygenation significantly, the optimal level of PEEP in children who have undergone the correction of various congenital heart defects through RVIAT is unclear, and additional research is needed to answer these questions. There are numerous other limitations inherent to the study, including the single-center design, the narrow age range, the brief follow-up period (2 h after CPB), and the absence of data on longer-term respiratory outcomes.

## 5. Conclusions

In summary, our findings provide some indication that the use of ARM improves pulmonary oxygenation and attenuates the systemic inflammatory response after extracorporeal circulation. In addition, ARM reduces the duration of postoperative mechanical ventilation and the incidence of pulmonary atelectasis in children.

## Figures and Tables

**Table 1:** Comparison of clinical characteristics and cardiopulmonary bypass duration data. Note: Data are expressed as medians (IQRs) or frequencies (%).

	Group C	Group A	*p*
Male/female	21/17	23/16	0.461
Age (yr)	1.5 ± 0.9	1.6 ± 1.1	0.664
Body mass (kg)	7.8 ± 1.2	8.2 ± 0.9	0.102
EF (%)	65.6 ± 9.5	68.1 ± 11.2	0.295
Pulmonary hypertension (mmHg)	48.2 ± 11.7	47.1 ± 9.9	0.657
OR time (min)	169.1 ± 18.5	171.6 ± 28.2	0.079
CPB time (min)	53.6 ± 5.1	53.1 ± 7.8	0.741
Aortic cross-clamp time (min)	30.2 ± 4.8	32.1 ± 5.1	0.097

**Table 2:** Number of postoperative complications. Note: Data are expressed as medians (IQRs) or frequencies (%). * Some patients had more than one pulmonary complication.

	C Group	A Group	*p* Value
Any pulmonary complication (n)	12/38 (31.6%)	5/39 (12.8%)	0.043
Type of pulmonary complication *			
Atelectasis	12/38 (31.6%)	4/39 (10.3%)	0.020
Pulmonary edema	0	2	0.253
Pneumonia	5	2	0.205
Pleural effusion	2	0	0.240
Acute lung injury	3	0	0.115
			
Ventilation (h)	4.2 ± 1.3	3.1 ± 1.5	0.003
ICU stay (h)	12.4 ± 1.7	11.5 ± 3.1	0.120
Postoperative hospital stay (d)	7.1 ± 1.6	6.0 ± 1.8	0.006
Mortality (n)	-	-	-

**Table 3:** Intraoperative proinflammatory cytokine levels. Note: Data are expressed as medians (IQRs).

		C Group	A Group	*p*
IL-6 (pg/mL)	T1	98.4 ± 16.8	98.7 ± 18.3	0.9405
	T4	138.8 ± 30.1	125.8 ± 25.1	0.043
TNF-α (pg/mL)	T1	119.6 ± 20.3	115.3 ± 21.9	0.375
	T4	176.8 ± 23.2	170.2 ± 29.3	0.278

**Table 4:** Hemodynamic data during the different experimental conditions. Note: Data are expressed as medians (IQRs). MBP = mean blood pressure; CI = cardiac index; HR = heart rate.

		C Group	A Group	*p* Value
CI (L/min/m^2^)	T1	2.7 ± 0.4	2.8 ± 0.6	0.394
	T2	2.8 ± 0.5	2.4 ± 0.5	0.001
	T3	2.9 ± 0.4	2.2 ± 0.3	0.001
	T4	2.8 ± 0.6	3.0 ± 0.6	0.148
MBP (mmHg)	T1	56.5 ± 8.9	55.8 ± 8.5	0.725
	T2	55.2 ± 10.1	50.6 ± 7.9	0.029
	T3	58.7 ± 9.6	53.3 ± 10.0	0.018
	T4	60.3 ± 9.9	60.9 ± 12.1	0.813
HR (beats/min)	T1	109.6 ± 11.9	108.3 ± 14.5	0.669
	T2	121.8 ± 21.4	123.3 ± 19.3	0.748
	T3	115.8 ± 16.9	117.6 ± 16.8	0.641
	T4	116.4 ± 19.8	114.3 ± 21.8	0.660

## Data Availability

If the application is approved, raw data will provided for a fee. We cannot provide access to the data, analytic methods, or research materials because Beijing Anzhen Hospital, Capital Medical University, holds the intellectual property rights to this article.

## Abbreviations

The following abbreviations are used in this manuscript:

VSD	ventricular septal defect
ASD	atrial septal defect
RVIAT	right vertical infra-axillary thoracotomy
ARM	alveolar recruitment maneuver
CPB	cardiopulmonary bypass
PEEP	positive end-expiratory pressure
CHD	congenital heart disease
